# Prediction of antenatal bleeding and preterm deliveries using placental magnetic resonance imaging in patients with placenta previa

**DOI:** 10.1007/s11604-024-01541-3

**Published:** 2024-02-19

**Authors:** Yuko Otake, Atsushi Ugajin, Hironori Takahashi, Yuya Tanaka, Hiroyuki Fujii, Mitsuru Matsuki, Harushi Mori

**Affiliations:** 1 Department of Radiology, Jichi Medical University, 3311-1 Yakushiji, Shimotsuke, Tochigi 329-0498 Japan; 2 Department of Radiology, National Hospital Organization Tochigi Medical Center, 1-10-37 Naka-Tomatsuri, Utsunomiya, Tochigi 320-8550 Japan; 3 Department of Obstetrics and Gynecology, Jichi Medical University, 3311-1 Yakushiji, Shimotsuke, Tochigi 329-0498 Japan; 4 Department of Radiology, The University of Tokyo, 7-3-1 Hongo, Bunkyo, Tokyo 113-8654 Japan

**Keywords:** MRI, Placenta previa, Hematoma, Antenatal bleeding, Preterm delivery

## Abstract

**Purpose:**

This study aimed to clarify associations between subacute hematoma on placental magnetic resonance imaging (MRI), antenatal bleeding, and preterm deliveries in patients with placenta previa (PP) without placenta accreta spectrum (PAS).

**Materials and methods:**

This retrospective study investigated 78 consecutive patients with PP (median age, 34.5 years; interquartile range [IQR], 31–37 years) who underwent placental MRI in the third trimester. Patients with PAS detected intraoperatively or pathologically were excluded. Two radiologists evaluated the presence of subacute hematomas and their locations on placental MRI. We examined associations between presence of subacute hematoma and antenatal bleeding, emergency cesarean section (CS), hysterectomy, gestational age (GA) at delivery, birth weight, and amount of blood loss at CS. We also examined the association between perinatal outcome and subacute hematoma location: marginal, retro-placental, or intra-placental. Inter-observer agreement for the detection of subacute hematoma was calculated using kappa analysis.

**Results:**

Subacute hematomas were identified on MRI in 39 of the 78 patients (50.0%). Antenatal bleeding and emergency CS were more prevalent in patients with subacute hematoma on MRI (20 patients [51.3%] and 18 patients [46.2%], respectively) than in patients without (7 patients [17.9%], Fisher’s exact test, *p* = 0.004 and 7 patients [17.9%], *p* = 0.014, respectively). GA at delivery was significantly lower in patients with subacute hematoma (median 36w3d, IQR 35w4d–37w1d) than in patients without (median 37w1d, IQR 36w4d–37w2d; Mann–Whitney test: *p* = 0.048). Marginal hematoma was significantly associated with antenatal bleeding and emergency CS. Inter-observer agreement for the presence of subacute hematoma was moderate (*κ* = 0.573).

**Conclusion:**

Subacute hematoma on placental MRI was associated with antenatal bleeding, emergency CS and shorter GA at delivery in patients with PP. Marginal hematoma was also associated with antenatal bleeding and emergency CS. Placental MRI appears useful for predicting antenatal bleeding and preterm delivery in patients with PP.

## Introduction

In placenta previa (PP), the placenta is located in the lower part of the uterus where uterine contractility is low and covers the internal os. Patients with PP are prone to antenatal bleeding related to remodeling of the placenta and cervix, and postpartum hemorrhage associated with poor contractility [1]. Moreover, antenatal bleeding in patients with PP is prone to repeat with increasing severity [1], which can necessitate preterm cesarean delivery, blood transfusion, and cause coagulopathy, hemodynamic instability, and multi-organ failure, and peripartum hysterectomy.

PP is most commonly evaluated using ultrasonography (US) in the third trimester of pregnancy to confirm its location, although magnetic resonance imaging (MRI) is sometimes utilized. MRI is also useful in the diagnosis of placenta accreta spectrum (PAS) associated with PP. Hematoma and infarction are often found in PAS [2–11], but can be observed even in patients without PAS [12–15]. However, the relationship between placental hematoma on MRI and perinatal outcome in patients with PP without PAS remains unclear. This study therefore investigated associations between subacute hematoma on placental MRI and prenatal outcomes in such patients.

## Materials and methods

### Patients

This retrospective study was approved by the institutional review board of our hospital (approval no. 20–164). The need to obtain informed consent was waived due to the retrospective nature of the study. Between October 16th, 2008, and May 31st, 2021, a total of 105 pregnant women with PP underwent MRI and cesarean section (CS) at our hospital. MRI was performed to determine the site of uterine incision (e.g., completely anterior placenta) or on suspicion of PAS from the presence of PP, surgical history, or ultrasonographic findings. All patients underwent MRI in the early third trimester. Experienced obstetricians defined the degree of PP as total, partial, or marginal when the placenta overlapped the internal ostium by ≥ 2, < 2 but > 0 cm, or 0 cm, respectively, according to the Japan Society of Obstetrics and Gynecology guidelines [16]. PP was diagnosed using transvaginal US immediately before delivery. To investigate PP without PAS, we excluded all 27 patients in whom PAS had been detected intraoperatively or pathologically (16 patients diagnosed with PAS during CS and 11 patients diagnosed with PAS on pathologic findings at hysterectomy) [17]. Ultimately, 78 patients were included in this study.

### Technique for MRI

All MRI examinations were performed using a 1.5-T scanner (Avanto and Symphony; Siemens Healthcare, Erlangen, Germany) with a torso phased-array coil. Obtained sequences included T2-weighted imaging (T2WI) with half-Fourier acquisition single-shot turbo spin echo (HASTE) (field of view [FOV], 280–300 mm; matrix, 256; repetition time [TR], 800–1000 ms; echo time [TE], 70–90 ms; flip angle, 20°) and true fast imaging with steady-state free procession (true FISP) (FOV, 280–300 mm; matrix, 256; TR, 540–800 ms; TE, 1.65–2.23 ms; flip angle, 70–80°) in three planes (axial, sagittal, and coronal), T1-weighted gradient echo imaging with fat suppression (FS-T1WI) using the Dixon technique (FOV, 400 mm; matrix, 256; TR, 7.04 ms; TE, 2.4/4.8 ms; flip angle, 15°) in one or more of three planes, including at least the sagittal plane. Each slice was 3–8 mm thick. No intravenous paramagnetic contrast material was administered.

### Assessment of placental hematoma

#### MRI

Two board-certified radiologists with 8 and 15 years of abdominal imaging experience, blinded to the perinatal outcome, independently assessed the presence and location of subacute hematoma in and around the placenta. Discrepancies in the judgments of the two radiologists were resolved by consensus decision.

**Fig. 1:**
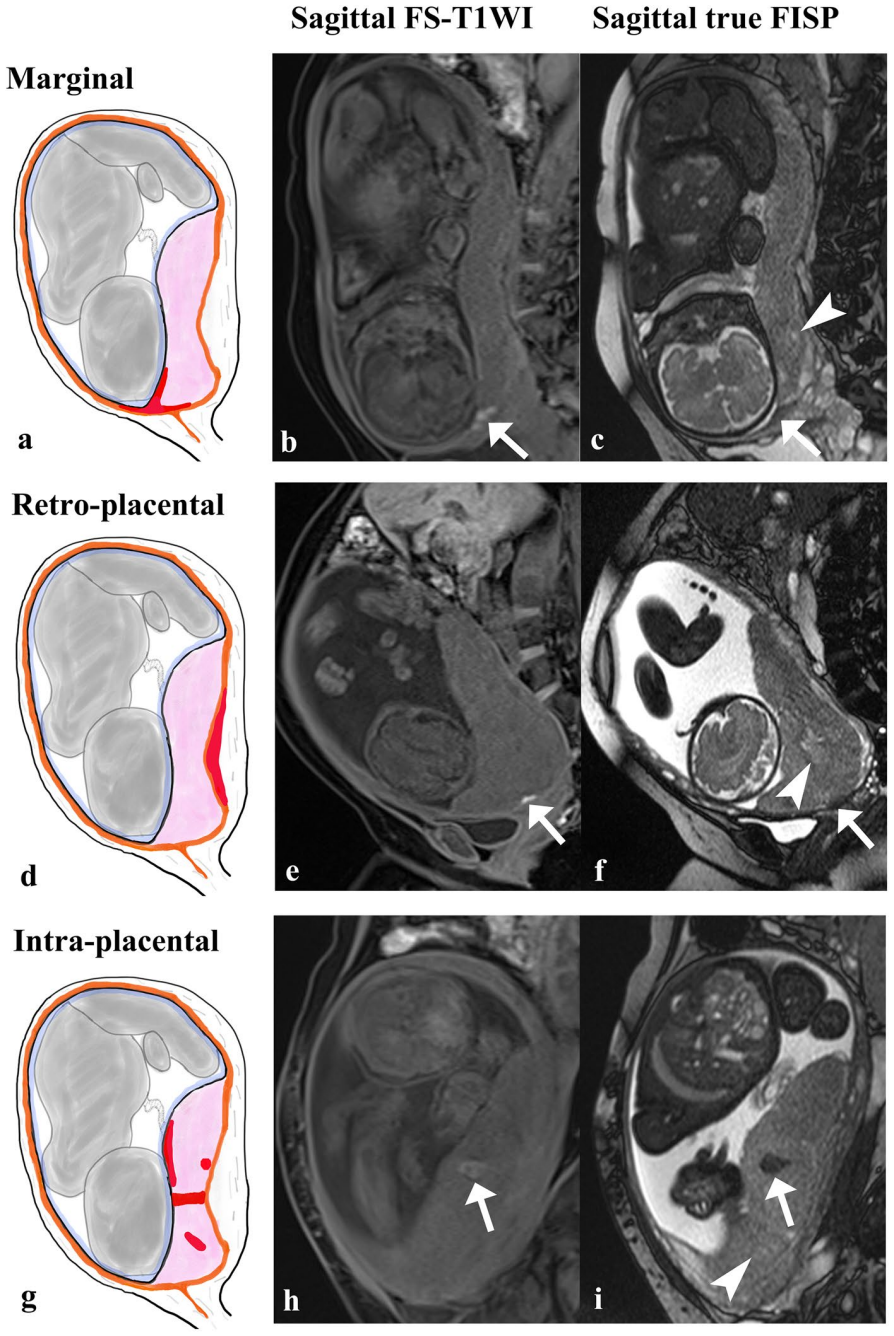
Classification of subacute hematoma by location, and representative placental magnetic resonance imaging of each location. Subacute hematomas (arrows) show hyperintensity on T1-weighted imaging with fat suppression (FS-T1WI) and hypointensity on true fast imaging with steady-state free precession image (true FISP). Cotyledons (arrowheads) are landmarks of the placental parenchyma. The pink areas in schematic illustrations (**a**, **d**, **g**) show the placenta and the red areas show the hematoma. **a–c** Marginal hematoma located at the placental margin. **d–f** Retro-placental hematoma located between the decidua membrane and the myometrium. The hematoma is not continuous with the placental margin. **g–i** Intra-placental hematoma is located in the subchorionic area of the placental bed, the fetal side, or intervillous.

We defined subacute hematoma as an area showing hyperintensity on FS-T1WI and hypointensity on true FISP, following previous reports [6, 18–22]. Because of its ability to quickly examine the entire uterus in three directions, true FISP was evaluated using T2WI instead of HASTE. To identify subacute hematoma, we first looked for hyperintensity in or around the placenta on FS-T1WI. Next, we detected the margin of the placenta while confirming cotyledons on true FISP to distinguish positioning relative to the placental parenchyma. Cotyledons are functional units and landmarks of the placental parenchyma, representing “round high-signal lesions delineated by a subtle peripheral low-signal line” on true FISP [23, 24]. Third, we looked for hypointensity on true FISP in the area where the FS-T1WI hyperintensity was present. Finally, the identified subacute hematomas were classified as marginal, retro-placental, or intra-placental hematoma based on their location on the placental MRI [2, 6, 22, 25]. We identified no anterior-placental hematomas. Representative images are shown in Fig. 1.

#### Pathology

We pathologically confirmed the placentas of patients from whom placental and/or uterine specimens were obtained and compared the results with MRI findings.

### Maternal characteristics and perinatal outcomes

We investigated the following maternal characteristics: age, gestational age (GA) at the time of MRI, degree of PP, parity, history of uterine surgery (prior CS history, dilatation and evacuation including curettage, myomectomy, adenomyomectomy, and endometrial polypectomy). We also investigated the following perinatal outcomes: antenatal bleeding after MRI, emergency CS, hysterectomy, blood transfusion, intrauterine balloon implantation, uterine artery embolization, GA at delivery and birth weight, and blood loss volume at CS. Amniotic volume was included in blood loss at CS. The number of days from MRI to delivery was also confirmed. We divided patients into two groups: patients with subacute hematoma on placental MRI; and patients without subacute hematoma on placental MRI. We then compared maternal characteristics and perinatal outcomes between groups. In addition, we evaluated the association between subacute hematoma location and maternal characteristics or perinatal outcomes.

### Statistical analysis

We performed univariate analysis using the Mann–Whitney and Fisher's exact tests to investigate the association between the presence and locations of subacute hematoma and maternal characteristics or perinatal outcomes. Continuous data are presented as median and interquartile range (IQR).

We evaluated inter-observer agreement between the two observers for the presence and the locations of subacute hematoma using kappa statistics, with values interpreted as follows: *κ* ≤ 0.20, none to slight agreement; *κ* = 0.21–0.40, fair agreement; *κ* = 0.41–0.60, moderate agreement; *κ* = 0.61–0.80, substantial agreement; and *κ*0.81–1.00, almost perfect agreement [26].

Statistical analysis was performed using SPSS version 28.0 (IBM, Armonk, NY). Values of *p* < 0.05 were considered significant.

## Results

### Assessment of placental hematoma

#### MRI

Table 1 shows the number of patients with subacute hematomas and their locations on placental MRI. Subacute hematoma was identified in 39 of the 78 patients (50.0%), with only marginal hematoma in 18, only retro-placental hematoma in 7, only intra-placental hematoma in 4, both marginal and retro-placental hematoma in 1, both marginal and intra-placental hematoma in 7, and retro-placental and intra-placental hematoma in 2. No patients showed hematoma in all three locations. Including overlapping hematoma locations, 26 hematomas were found at the placental margin, 10 retro-placentally, and 13 intra-placentally. All marginal hematomas were found near the internal os.

**Table 1:** Number of patients with subacute hematomas and hematoma locations on placental MRI

	Total	Location of subacute hematoma					
		Marginal hematoma	Retro-placental hematoma	Intra-placental hematoma	Marginal + retro-placental hematoma	Marginal + intra-placental hematoma	Retro + intra-placental hematoma
Number of patients	39	18	7	4	1	7	2

*MRI* magnetic resonance imaging

#### Pathology

The placenta was pathologically examined in 36 of the 78 patients, comprising 7 patients who had undergone hysterectomy and 29 for whom the placenta alone was examined. In the group with subacute hematoma on MRI, the placentas of 19 patients were examined pathologically and 4 abnormalities were found: one patient each had retro-placental hematoma, both intra-placental thrombosis and placental infarction, both retro-placental hematoma and intra-placental thrombosis, and placental infarction. In the group without hematoma, 17 placentas were examined pathologically and 3 abnormalities were found: one patient each had retro-placental hematoma, intra-placental thrombosis, and both intra-placental thrombosis and placental infarction.

### Maternal characteristics and perinatal outcomes

Table 2 shows maternal characteristics and perinatal outcomes of patients with and without subacute hematoma on placental MRI. Both total PP and history of CS were more prevalent in patients with subacute hematoma than in those without subacute hematoma (33 versus 22 patients, *p* = 0.012, 15 versus 7 patients: *p* = 0.005, respectively). Antenatal bleeding occurred in 27 (34.6%) and emergency CS was needed in 25 (32.1%) of the 78 patients, and both were more prevalent in patients with subacute hematoma (20 versus 7 patients, *p* = 0.004, and 18 versus 7 patients, *p* = 0.014, respectively). GA at delivery was also shorter in patients with subacute hematoma (median 36w3d [IQR 35w4d–37w1d] versus 37w1d [IQR 36w1d–37w2d], *p* = 0.048).

**Table 2:** Maternal characteristics and perinatal outcomes of patients with and without subacute hematoma on placental MRI

Maternal characteristics	Total (*n* = 78)	With subacute hematoma (*n* = 39)	Without subacute hematoma (*n* = 39)
Age (years), median (IQR)	34.5 (31–37)	35 (33–37)	34.5 (30.5–36.5)
GA at MRI, median (IQR)	33w5d (32w3d–34w4d)	33w1d (32w2d–34w2d)	33w1d (32w2d–34w2d)
Degree of PP			
Total,* n* (%)	55 (70.5)	33 (84.6)^*1^	22 (56.4)
Partial/marginal,* n* (%)	23 (29.5)	6 (15.4)	17 (43.6)
Primiparas,* n* (%)	22 (28.2)	8 (20.5)	14 (35.9)
History of uterine surgery			
CS,* n* (%)	22 (28.2)	15 (38.5)^*2^	7 (17.9)
Myomectomy,* n* (%)	5 (6.4)	1 (2.6)	4 (10.2)
D&E,* n* (%)	18 (23.1)	8 (20.5)	10 (25.6)
Adenomyomectomy,* n* (%)	1 (1.3)	0 (0)	0 (0)
Endometrial polypectomy,* n* (%)	1 (1.3)	0 (0)	1 (2.6)
Perinatal outcomes			
Antenatal bleeding,* n* (%)	27 (34.6)	20 (51.3)^*3^	7 (17.9)
Emergency CS,* n* (%)	25 (32.1)	18 (46.2)^*4^	7 (17.9)
Hysterectomy,* n* (%)	7 (9.0)	4 (10.3)	3 (7.7)
Transfusion,* n* (%)	52(66.7)	27 (69.2)	25 (64.1)
Autologous,* n* (%)	48 (61.5)	24 (61.5)	24 (61.5)
Homologous,* n* (%)	13 (16.7)	7 (17.9)	6 (15.4)
Intrauterine balloon* n* (%)	36 (46.2)	18 (46.2)	18 (46.2)
Uterine artery embolization,* n* (%)	1 (1.3)	1 (2.6)	0 (0)
GA at delivery, median (IQR)	36w6d (36w0d–37w2d)	**36w3d (35w4d**–37w1d)^*5^	37w1d (36w1d–37w2d)
Birth weight (g), median (IQR)	2652 (2407–2931)	2604 (2405.5–2787.5)	2748 (2445.5–3014)
Blood loss at CS (ml), median (IQR)	1335 (965–1900)	1320 (950–1745)	1350 (1000–1900)
Days from MRI to delivery(d), median (IQR)	19.5d (15d–28d)	20d (16.5d–27.5d)	18d (11.5d–29d)

*P* values were obtained for comparisons between presence and absence of subacute hematoma and each characteristic or outcome using the Mann–Whitney test or Fisher's exact test

*MRI* magnetic resonance imaging, *n* number, *IQR* interquartile range, *GA* gestational age, *w* weeks, *d* days, *PP* placenta previa, *CS* cesarean section, *D&E* dilatation and evacuation

*Significant difference (*p* = 0.012^*1^, 0.005^*2^, 0.004^*3^, 0.014^*4^, 0.048^*5^, respectively) are indicated in bold

Seven of the 78 patients underwent hysterectomy unrelated to the presence of placental hematoma on MRI. Four patients with subacute hematoma on MRI underwent hysterectomy: two for atonic bleeding (one with intra-placental hematoma only; one with both marginal and intra-placental hematoma), one for asymptomatic uterine rupture (with only retro-placental hematoma) and one for disseminated intravascular coagulation (with marginal and intra-placental hematomas). Three patients without subacute hematoma underwent hysterectomy for atonic bleeding.

Table 3 shows maternal characteristics and perinatal outcomes of patients by placental hematoma without overlapping locations on MRI. Here, only groups with only one hematoma location on MRI were evaluated. Patients with only marginal hematomas showed a higher prevalence of antenatal bleeding (10 versus 7 patients, *p* = 0.002), emergency CS (9 versus 7 patients, *p* = 0.008), and shorter GA at delivery (36w3d [IQR 35w5d–37w0d] vs 37w1d [IQR 36w1d–37w2d], *p* = 0.039) compared to those without subacute hematoma. No association with perinatal outcome was demonstrated for retro- or intra-placental hematoma.

**Table 3:** Maternal characteristics and perinatal outcomes by placental hematoma without overlapping location on MRI

Maternal characteristics	Marginal hematoma (*n* = 18)	Retro-placental hematoma (*n* = 7)	Intra-placental hematoma (*n* = 4)
Age (years), median (IQR)	34 (31–36)	34 (34–37)	36(33.75–36.25)
GA at MRI, median (IQR)	33w2d (32w1d–34w6d)	33w0d (32w2d–34w4d)	34w0d (33w4d–34w3d)
Degree of PP			
Total PP,* n* (%)	15(83.3)	7 (100)	4 (100)
Partial/marginal PP,* n* (%)	3 (16.6)	0 (0)	0 (0)
Primiparas,* n* (%)	1 (5.6)	3 (42.9)	2 (50)
History of uterine surgery			
CS,* n* (%)	7(38.9)	3 (42.8)	0 (0)
Myomectomy,* n* (%)	0 (0)	0 (0)	0 (0)
D&E,* n* (%)	4 (22.2)	1 (14.2)	1 (25)
Adenomyomectomy,* n* (%)	0 (0)	0 (0)	0 (0)
Endometrial polypectomy,* n* (%)	0 (0)	0 (0)	0 (0)
Perinatal outcomes			
Antenatal bleeding,* n* (%)	10 (55.6)^*1^	1 (14.3)	3 (75)
Emergency CS,* n* (%)	9 (50)^*2^	1 (14.3)	2 (50)
Hysterectomy,* n* (%)	0 (0)	1 (14.3)	1 (25)
Transfusion,* n* (%)	9 (50)	5 (71.4)	3 (75)
Autologous,* n* (%)	9 (50)	4 (57.1)	3 (75)
Homologous,* n* (%)	0 (0)	2 (28.6)	1 (25)
Intrauterine balloon* n* (%)	6 (33.3)	5 (71.4)	2 (50)
Uterine artery embolization,* n* (%)	0 (0)	0 (0)	0 (0)
GA at delivery, median (IQR)	**36w3d (35w5d**–37w0d)^*3^	37w0d (36w5d–37w1d)	36w2d (35w3d–37w5d)
Birth weight (g), median (IQR)	2606 (2446–2698)	2472 (2272–2741)	2491 (2391.5–2655.5)
Blood loss at CS (ml), median (IQR)	1300 (1050–1580)	850(685–1605)	1387.5 (913.75–2547.5)
Days from MRI to delivery (d), median (IQR)	20 (11–29)	21 (17–28.5)	14.5 (10.75–19.5)

*P* values were obtained for comparisons between each hematoma location and each characteristic or outcome using the Mann–Whitney test or Fisher's exact test

*MRI* magnetic resonance imaging, *n* number, *IQR* interquartile range, *GA* gestational age, *w* weeks, *d* days, *PP* placenta previa, *CS* cesarean section, *D&E* dilatation and evacuation

* Significant difference (*p* = 0.002^*1^, 0.008^*2^, 0.039^*3^, respectively) are indicated in bold

Table 4 shows maternal characteristics and perinatal outcomes of patients with marginal hematoma, including overlap with retro-placental or intra-placental hematoma on MRI. Patients with marginal hematoma with overlapping locations on MRI also showed a higher prevalence of antenatal bleeding (14 versus 7 patients, *p* = 0.003), emergency CS (13 versus 7 patients, *p* = 0.006) compared to those without subacute hematoma. But no significant association with shorting of GA at delivery was demonstrated (*p* = 0.065). One patient with both marginal and intra-placental hematoma on MRI needed uterine artery embolization and hysterectomy, and another patient with marginal and intra-placental hematoma on MRI needed hysterectomy. Pathological specimens of the placenta were obtained in both patients, but no hematoma or other abnormalities were identified.

**Table 4:** Maternal characteristics and perinatal outcomes of patients with marginal hematoma, including overlap with retro-placental or intra-placental hematoma on MRI

Maternal characteristics	Marginal hematoma overlapping with retro-placental or intra-placental hematoma (*n* = 26)
Age (years), median (IQR)	34 (32–37)
GA at MRI, median (IQR)	33w2d (32w2d-34w1d)
Degree of PP	
Total PP,* n* (%)	20 (76.9)
Partial/marginal PP,* n* (%)	6 (23.1)
Primiparas,* n* (%)	3 (11.5)
History of uterine surgery	
CS,* n* (%)	12(46.1)
Myomectomy,* n* (%)	1 (3.8)
D&E,* n* (%)	5 (19.2)
Adenomyomectomy,* n* (%)	0 (0)
Endometrial polypectomy,* n* (%)	0 (0)
Perinatal outcomes	
Antenatal bleeding,* n* (%)	14 (53.8)^*1^
Emergency CS,* n* (%)	13 (50)^*2^
Hysterectomy,* n* (%)	2^a^ (7.7)
Transfusion,* n* (%)	16 (61.5)
Autologous,* n* (%)	14 (53.8)
Homologous,* n* (%)	4 (15.3)
Intrauterine balloon* n* (%)	10 (38.5)^*3^
Uterine artery embolization,* n* (%)	1^a^ (3.8)
GA at delivery, median (IQR)	36w3d (35w5d-37w2d)
Birth weight (g), median (IQR)	2652 (2446–2698)
Blood loss at CS (ml), median (IQR)	1300 (1050–1600)
Days from MRI to delivery (d), median (IQR)	18 (11–29)

*P* values were obtained for comparisons between each hematoma location and each characteristic or outcome using the Mann–Whitney test or Fisher's exact test

*MRI* magnetic resonance imaging, *n* number, *IQR* interquartile range, *GA* gestational age, *w* weeks, *d* days, *PP* placenta previa, *CS* cesarean section, *D&E* dilatation and evacuation

*Significant difference (*p* = 0.003^*1^, 0.006^*2^, 0.016^*3^, respectively) are indicated in bold

^a^One patient with both marginal and intra-placental hematoma needed uterine artery embolization and hysterectomy, and another patient with marginal and intra-placental hematoma needed hysterectomy

Pathological placental abnormality was not associated with any perinatal outcome in 36 patients with confirmed placental pathology, nor in the group with subacute hematoma on MRI (17 patients). However, all four patients with subacute hematoma on MRI had antenatal bleeding; three of the four patients with subacute hematoma on MRI experienced emergency CS and preterm delivery before 37 weeks, including one who also required hysterectomy. Three patients without a subacute hematoma on MRI did not have these perinatal outcomes.

### Inter-observer agreement

Inter-observer agreement was moderate for the presence of subacute hematoma (*κ* = 0.573). Inter-observer agreement was moderate to fair when including overlapping hematoma locations (marginal, *κ* = 0.496; retro-placental, *κ* = 0.428; intra-placental, *κ* = 0.316), but slight for hematoma of each location when not including overlap (*κ* < 0.20, respectively).

## Discussion

Our study showed that subacute hematoma placental MRI was associated with antenatal bleeding, emergency CS and shorter GA at delivery in PP. We also showed that marginal hematoma was associated with antenatal bleeding and emergency CS.

Marginal hematomas were the most common, both alone and when overlapping. Marginal hematomas differ from subchorionic hematoma, which is frequently seen in the first trimester [27–31]. Marginal hematoma in this study showed subacute-phase signals that are detected around 1 day to 2 weeks after bleeding [6, 18–22]. These hematomas were formed in the late second trimester or later, because all MRI examinations were performed in the early third trimester. Although we cannot explain why subacute hematomas were more common at the margin than at other locations of the placenta, venous sinuses may be associated with hematoma formation. These form at the placental margins and are prone to hemorrhage [32, 33]. In particular, in marginal PP, these sinuses are located near the internal os and antenatal bleeding and subsequent emergency CS are known to occur more frequently than in total PP [32]. Although further studies are needed, marginal hematoma on MRI may provide a predictor of subsequent preterm delivery following antenatal bleeding.

Retro-placental hematoma showed no association with perinatal outcomes. Retro-placental hematoma suggests placental abruption [21, 22, 25, 28], which is a major cause of antenatal bleeding in the 3rd trimester of pregnancy and is associated with perinatal outcomes, including preterm delivery [1, 21, 34]. In this study, MRI could not be performed on unstable patients with large posterior placental hematomas considered to represent placental abruption. This may be one reason why retro-placental hematoma showed an association with perinatal outcomes in our study.

We also could not clarify the association between preterm delivery and intra-placental hematoma, which is known to occur due to vascular, toxic, degenerative, and infectious damage and has been reported as a predictor of preterm delivery and miscarriage [12, 13, 27, 28]. This may be due to the small number of patients with only intra-placental hematoma.

Statistically, pathological placental abnormality was not associated with any perinatal outcomes. However, three of the four patients had both pathological placental abnormalities and subacute hematomas on MRI also had prenatal hemorrhage, emergency CS, and preterm delivery. In these patients, placental abnormalities may have been present from the time of MRI to delivery, which is consistent with the need for early delivery [28]. Because there were only a few patients in which both pathology and MRI proved placental abnormalities, we believe that we need to accumulate more cases in future.

Inter-observer agreement for the presence of subacute hematoma was relatively good. The present results suggest that the detection of subacute hematoma in patients with PP by FS-T1WI hyperintensity is reproducible and clinically useful, independent of the observer. Subacute hematoma is prominently recognized as a positive finding, particularly when fat suppression is applied. Reports about placental abruption [21] and PAS [10, 11] have shown the utility of T1WI in diagnosing hematomas, and combination with fat suppression is also recommended [11]. On the other hand, good agreement was not obtained for each hematoma location without overlap. This may be because the T1WI hyper-intensities of subacute hematomas were sometimes unclear or heterogeneously distributed, making it difficult to pick up or classify the hematoma location. In particular, it was difficult to classify subacute hematomas as marginal hematomas when the location of the heterogeneous T1WI hyper-intensity within the hematoma was close to the posterior placental wall. In such cases where there was a discrepancy in the judgments of the two radiologists, the overall distribution of the hematoma was confirmed together by the two radiologists and defined it as a marginal hematoma.

Compared to US, MRI offers the advantage of providing a wider, clearer field of view independent of the observer, contributing to the present results. This modality is useful in the evaluation of placenta involving the posterior uterine wall (posterior placenta), structure of the uterus, location of the placenta, and external surface of the uterus [21, 22, 25, 35]. MRI is known to be particularly useful in detecting retro-placental hematoma (abruption) with posterior placenta, which is difficult with US [20]. Takahashi et al. reported that patients with lateral PP experienced significantly less bleeding than those with non-lateral PP when placenta location was classified according to MRI [36]. Nagase et al. reported that the identification of posterior extrauterine adhesion on MRI was associated with increased intraoperative bleeding in patients with PP [37]. Thus, MRI is another useful tool alongside US for assessing prognosis and the need for blood transfusion or early CS in patients with PP.

Several limitations to this study need to be considered. First, when PP is complicated by PAS, determining the origin of the abnormal findings is difficult. Second, comparison of MRI findings and pathology specimens was not sufficient. Placental and uterine pathologies could not be confirmed in all patients, and it was not possible to compare the location of pathological abnormalities with the location of abnormal findings on MRI. And pathologies were also difficult to verify from pathological specimens because a gap of about 20 days existed between MRI and delivery and perinatal hematoma was often lost during delivery. Third, the association between hematoma volume and perinatal outcome was not evaluated in this study. An association between hematoma volume and grade of abruption has been suggested [12, 21], but a report on infant neuromotor developmental and placental vasculopathies showed no associations with lesion volume [15]. These limitations represent potential subjects for future study.

In conclusion, subacute hematoma on placental MRI was associated with antenatal bleeding, emergency CS, and shorter GA at delivery in PP. We also showed that marginal hematoma was associated with antenatal bleeding and emergency CS. Placental MRI appears useful for predicting antenatal bleeding and preterm delivery in PP.
